# Gut metabolomic profiles in paediatric ulcerative colitis patients prior to and after receiving faecal microbiota transplants

**DOI:** 10.1017/gmb.2023.15

**Published:** 2023-10-06

**Authors:** Parastou S. Khalessi Hosseini, Beibei Wang, Yihui Luan, Fengzhu Sun, Sonia Michail

**Affiliations:** 1 Los Angeles County – University of Southern California, Los Angeles, CA, USA; 2 School of Mathematics, Shandong University, Jinan, China; 3 Quantitative and Computational Biology and Mathematics, University of Southern California, Los Angeles, CA, USA; 4 Gastroenterology, Children’s Hospital of Los Angeles, Los Angeles, CA, USA

**Keywords:** metabolomics, inflammatory bowel disease, ulcerative colitis, fecal microbiota transplant

## Abstract

Ulcerative colitis (UC) is an immune-mediated inflammation of the colonic mucosa. Gut microbiota dysbiosis may play a significant role in disease pathogenesis by causing shifts in metabolomic profiles within the gut. To identify differences and trends in the metabolomic profile of paediatric UC patients pre- and post-faecal microbiota transplants (FMT). Forty-six paediatric patients with mild-to-moderate UC and 30 healthy paediatric patients were enrolled in this study. Baseline stool samples were collected prior to FMT initiation and at months 1, 3, 6, and 12 post-FMT. Pediatric Ulcerative Colitis Activity Index (PUCAI) scores were calculated at baseline and months 1, 3, 6, and 12 after FMT. The average Bray–Curtis dissimilarities to healthy subjects decreased after FMT. In principal coordinate analysis plots, UC patients’ centroids drew nearer to healthy individuals. The variance explained by phenotype (Healthy versus UC) reduced and remained significant. From 1 to 3 months after FMT, PUCAI trends were statistically significant and decreasing. PUCAI scores remain flat starting 6 months after FMT. This study concludes that paediatric UC patients have a significantly different baseline metabolite profile than healthy controls. Although being time limited, FMT significantly altered these metabolite profiles and shifted them towards that of healthy controls.

## Introduction

Ulcerative colitis (UC) is a type of inflammatory bowel disease (IBD) characterized by chronic immune-mediated intestinal inflammation of the colonic mucosal layer. Approximately 20-30% of patients become symptomatic and are diagnosed with UC before 18 years of age (Abraham, 2012). This early presentation can increase the risk of long-term physical and psychological sequelae in affected paediatric patients.

There is significant interest in the role of the colonic microbiota in both UC pathogenesis and management. It is hypothesized that dysbiosis within the gut can shift metabolite profiles and cause an imbalance between anti- and pro-inflammatory mediators. An interplay between these metabolites, genetics, and the environment could be an inciting factor and impact the disease course. Research continues to be limited on this topic and is primarily centred around adult UC patients. Given their unique and more severe disease course, paediatric-specific research is needed, as this could reflect differences in metabolite profiles (Tamboli et al., 2004; Lepage et al., 2008; Gever et al., 2014; Scoville et al., 2018).

Metabolite profiles can reflect the gut microbiome, as many metabolites are by-products of microbiome metabolism. For instance, UC patients with reduced *Ruminococcaceae* within the gut commonly have reduced levels of lithocholic acid and deoxycholic acid (Sinha et al., 2020). A metabolite’s protective versus detrimental role is determined by complex host-microbe interaction and different regulatory pathways (Diab et al., 2019; Shores et al., 2011; Staley et al., 2017).

Faecal microbiota transplants (FMT) could be a potential therapeutic option in paediatric UC patients by reducing dysbiosis and shifting the colonic ecosystem towards healthy donor levels. However, currently, there is a gap in knowledge of the baseline metabolite profiles of paediatric UC patients, as well as how these profiles change following FMT. This study aims to help define the paediatric UC patient’s metabolomic profile and describe individual metabolite trends post-FMT.

## Method

### Materials

#### Donors

The universal donor subject is an identified healthy volunteer ≥16 to ≤21 years old, has a BMI >18.5 and <25, has not been diagnosed with any chronic illness, is on a regular diet, and has not been taking any prescription, over-the-counter therapies, or probiotics for at least 3 months.

The universal donor subject has a negative serum HIV, Hepatitis A, B (Relman et al., 2013; Davidovics et al., 2019) and C, syphilis, and negative stool studies for culture, multidrug-resistant organisms, ova and parasites, Clostridium difficile, Giardia, and Cryptosporidium in accordance with recent guidelines endorsed by the American Gastroenterological Association (Bakken et al., 2011; Owens et al., 2013).

### Sample population

#### Inclusion criteria

Paediatric patients who have been diagnosed with mild-to-moderate ulcerative colitis. Mild-to-moderate disease was based on a Paediatric Ulcerative Colitis Activity Index (PUCAI) score of 10-64.

#### Exclusion criteria

Children with known resistance to steroid therapy, immunomodulators, and biologics or on a steroid dose greater than 0.5 mg/kg/day. Additionally, any child with a recent dose change in medications, allergy or intolerance to mesalamine or 5-ASA products, evidence of infectious colitis, a concurrent infection that required anti-microbial therapy, recently received probiotic preparations, recent or current pregnancy, currently breastfeeding, renal or liver dysfunction, congenital or acquired immunodeficiency due to conditions other than ulcerative colitis, recently received chemotherapy, recent diagnosis with HIV, or inability to give informed consent/assent.

### FMT preparation and administration

Heterologous (faeces from a healthy donor transplanted into a person with ulcerative colitis) and autologous (collection of faeces during patient’s healthy state for later use) were collected. All heterologous and autologous stool samples were collected on-site, transported on ice, and processed within two hours. The filtered healthy human donor subject stool solution was homogenized in sterile normal saline. Fifty grams of processed faecal material was infused into the terminal ileum through the working channel of the instrument during colonoscopy to allow delivery of the transplanted microbiome to the entire colon.

### Sample collection

Baseline stool samples from 44 different patients were collected prior to the administration of FMT. Follow-up stool samples were collected at months 1, 3, 6, and 12 after receiving one infusion of FMT. Stools were immediately collected, transported on ice, and placed into a -80C freezer until profiling.

### PUCAI scoring

PUCAI scores were calculated at baseline and at months 1, 3, 6, and 12 post-FMT.

### COVID-19 pandemic

All samples were collected prior to COVID-19 pandemic.

### Metabolomic profiling

Faecal samples were analyzed at the UC Davis West Coast Metabolomics Center using untargeted metabolomics by gas chromatography-time of flight-mass spectrometry (GC-TOF-MS). Metabolites were identified by comparison to the BinBase database (Nusbaum et al., 2018). Signal intensities were obtained for 230 metabolites which were included in subsequent analyses.

### Statistical analysis

Data analysis was performed in R version 4.1.2 (R Core Team, 2021). Bray–Curtis dissimilarities (Bray and Curtis, 1957) for the metabolomic data were calculated using “vegdist” function in the “vegan” package (version 2.5.7) (Oksanen et al., 2020) after normalizing the metabolomic profiles to relative abundances. A principal coordinate analysis (PCoA) (Dray et al., 2006) based on Bray–Curtis dissimilarities was implemented using the “pcoa” function in the “ape” package (version 5.6.2) (Paradis and Schliep, 2019) to project samples into two-dimensional Euclidean space. Quantifications of variance explained by different variables were calculated using permutational multivariate analysis of variance (PERMANOVA) (Anderson, 2001) with the “adonis” function in the “vegan” package (version 2.5.7) (Oksanen et al., 2020) based on Bray–Curtis dissimilarities. To avoid problems related to variable ordering, the total variance explained by each variable was evaluated independently of other variables and thus should be regarded as the total variance explainable by that variable (Lloyd-Price et al., 2019). The corresponding significances were assessed using permutational tests with 1,000 permutations.

Differential abundance analysis of metabolites was conducted by MaAsLin 2 (Microbiome Multivariable Associations with Linear Models) (Mallick et al., 2021). It provided a coherent paradigm through a multi-model framework with arbitrary coefficients and contrasts of interest and had been shown to produce more consistent results across different datasets (Nearing et al., 2022). Metabolites with a very low variance across all samples (below half the median of all feature-wise variances) were removed from the following analysis. We entered log-transformed metabolomic profiles into the primary “Maaslin2” function within the “MaAsLin2” R package (version 1.8.0) (Mallick et al., 2021) and then fit a linear model for each feature. Benjamini-Hochberg false discovery rate (BH-FDR)-corrected p values by Wald test were produced.

1. For identifying the differentially expressed metabolites between Healthy controls and UC cases at different time points after FMT, log-transformed abundances were fit with the following per-feature linear fixed-effects model: Feature ~ (intercept) + phenotype + gender + age + ethnicity, (1) where phenotype (Healthy/UC with Healthy as the reference group), gender (female/male), and ethnicity (Hispanic/non-Hispanic) were category variables, and age was a continuous variable. Significant associations were defined as those with BH-FDR q value of the corresponding coefficient below the threshold of 0.05.
2. For recognizing the differentially expressed metabolites between any two of the time points after FMT of UC cases, log-transformed abundances were fit with the following per-feature linear mixed-effects model: Feature ~ (intercept) + time point + age + gender + ethnicity + pancolitis + Clostridium difficile infection (CDI) history + FMT type + medication + (1|subject), (2) where the subject was included as a random effect to account for the correlations in the repeated measures. Time point (any pairs of the combinations of baseline, 1 month after FMT, 3 months after FMT, 6 months after FMT, 12 months after FMT, with the previous time point as the reference group), gender (female/male), ethnicity (Hispanic/non-Hispanic), pancolitis (no/yes), CDI history (no/yes), FMT type (autologous/heterologous), and medication (no/yes) were category variables. And age was a continuous variable. Significant associations were defined as those with BH-FDR q value of the corresponding coefficient below the threshold of 0.05.
3. In Figure 3, time points, CDI history, medication, ethnicity, and FMT type explained a significant variance of metabolites in UC cases. To figure out the influence of these confounding factors in the FMT process for UC cases, we fit the following per-feature linear fixed-effects model within each time point. Medications were not involved in the analysis due to their complicated prescriptions; however, patients receiving concurrent antimicrobial medications, probiotics, received or are receiving chemotherapy were excluded from the study. Feature ~ (intercept) +ethnicity + CDI history + FMT type, (3) where ethnicity (Hispanic/non-Hispanic), CDI history (no/yes), and FMT type (autologous/heterologous) were category variables. Significant associations were defined as those with BH-FDR q value of the corresponding coefficient below the threshold of 0.25.

## Results

### Demographics

Key patient information and demographics can be found in Table 1. In total, we included 30 healthy children and 46 paediatric UC patients. UC patients received FMTs. In this paper, we focussed on metabolomic profiles for UC patients at 5 different time points, including baseline (before FMT), 1 month after FMT, 3 months after FMT, 6 months after FMT, and 12 months after FMT. The average age of 30 healthy children was approximately 14 years, while paediatric UC patients were approximately 16 years of age. There were differences between the ethnicities for healthy and UC subjects. As shown in Table 1, all healthy individuals were non-Hispanics, while 52% of UC patients were non-Hispanics.

**Table 1.** Demographics of paediatric healthy controls and UC cases.

	Healthy	Ulcerative colitis
Number of patients	30	46
Age (average)	14.43	16.41
Gender (males)	14	27
Ethnicity (Hispanic)	0	22
Pancolitis (yes)	NA	30
Number of patients having CDI history	NA	23
Number of patients having autologous FMT	NA	12
Endoscopic Mayo score (average)	NA	2.26
Number of patients taking medications	NA	42
Average BMI	NA	22

Of the children with UC, 30 out of 46 patients had pancolitis, and half had CDI histories. 12 paediatric UC patients received autologous FMT, while the rest received heterologous FMT. In addition, 42 out of 46 patients received one or several therapies before FMT, such as proton pump inhibitors, biologic therapy, immunomodulators, 5-aminosalicylates, and antibiotics. For simplicity, we considered whether they were taking medications or not.

### Donor microbiota profile

**Figure 1.**
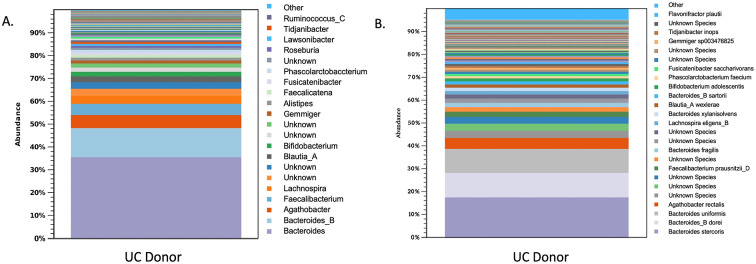
Shows the microbiota profile of donors by (A.) genus and (B.) species.

The microbiota had a Shannon diversity index of around 6.5, with the dominant microbiota consisting of *Firmicutes and Bacteriodetes.* There was also an abundance of *Faecalibacterium prausnitzii,* lactobacilli*, Bacteriodetes, and Bifidobacterium* (Figure 1).

### Metabolomes of paediatric UC patients shifted towards healthy profiles after FMT

**Figure 2.**
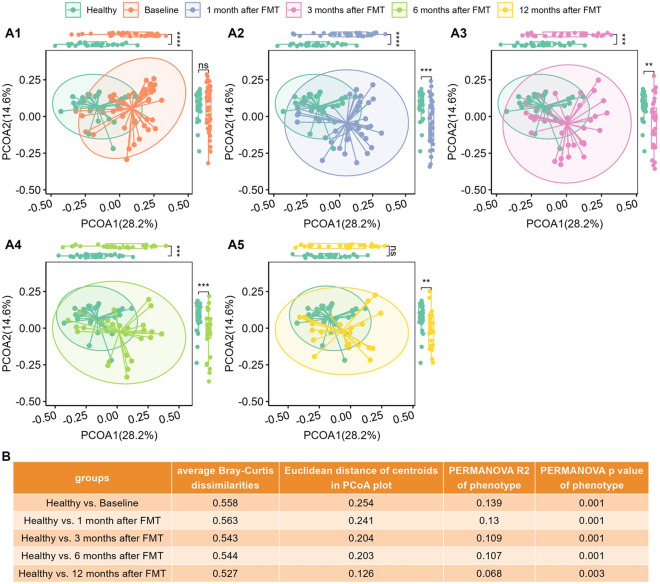
The metabolomic profiles of UC patients progressed to healthy levels after FMT. (A1)–(A5), PCoA plots based on metabolomics (Bray–Curtis dissimilarities on relative abundance) for Healthy controls versus UC cases at Baseline (A1), Healthy controls versus UC cases at 1 month after FMT (A2), Healthy controls versus UC cases at 3 months after FMT (A3), Healthy controls versus UC cases after 6 months after FMT (A4), and Healthy controls versus UC cases after 12 months after FMT (A5), separately. Box plots of PCoA and PCoA2 were shown in the margins of PCoA plots. Wilcoxon rank sum tests were used to compare the differences between Healthy and UC subjects at different time points after FMT, with ns (not significant) for *p* > 0.05, * for *p* <= 0.05, ** for *p* <= 0.01, *** for *p* <= 0.001, and **** for *p* <= 0.0001. (B) Quantitative differences between Healthy and UC subjects at different time points, including average Bray–Curtis dissimilarities between two groups, Euclidean distance between centroids of two groups in PCoA plots (A1)–(A5), variance explained (R2) by phenotype (Healthy versus UC) and respective p-value determined by PERMANOVA on metabolomics (Bray–Curtis distance on relative abundance).

We computed the Bray–Curtis distance between any pair of samples and used two-dimensional PCoA plots to visualize the samples based on metabolomic profiles. Figure 2 A1 shows that Healthy and UC baseline samples clustered into two groups according to their phenotypes.

Wilcoxon tests revealed that PCoA1 of UC patients significantly differed from that of healthy individuals (Figure 2A1), although PCoA2 did not vary statistically because of overdispersion (Figure 2AI).

Figure 1B shows that the average Bray–Curtis dissimilarities, the Euclidean distances between the centroids, and the variance explained by different groups between the healthy controls and the UC patients decrease with time after FMT. The last column of Figure 1C shows that although still significant, the variance explained by phenotype (Healthy versus UC) was reduced. Figure 2 clearly shows that metabolomes of paediatric UC patients shifted towards healthy profiles after FMT.

### Metabolite changes in paediatric UC patients

We next used a linear fixed-effects model (equation [1]) by MaAsLin 2 to identify metabolites associated with paediatric UC. A total of 230 metabolites were tested and metabolites with BH FDR less than 0.05 are shown in Figure 2. Among the differentially abundant metabolites, 10 amino acids (aminomalonate, cysteine, glutamine, leucine, n-acetylputrescine, n-epsilon-trimethyllysine, phenylalanine, tryptophan, tyrosine, and valine), 2- carboxylic acid (phenol and pyruvic acid), 1 fatty acid (arachidonic acid), and 7 miscellaneous (2-hydroxybutanoic acid, creatinine, glucuronic acid, lactic acid, myo-inositol, threonic acid, and urea) were significantly higher in UC patients compared to healthy individuals. While 1 amino

acid (epsilon-caprolactam), 2 bile acids (deoxycholic acid and lithocholic acid), 1 carboxylic acid (pipecolinic acid), 11 fatty acids (2-methylglutaric acid, 3-hydroxypalmitic acid, arachidic acid, heptadecanoic acid, lignoceric acid, myristic acid, nonadecanoic acid, octadecanol, palmitic acid, pentadecanoic acid, and stearic acid), 7 miscellaneous (6-deoxyglucose, 6-hydroxynicotinic acid, biphenyl, dehydrocholesterol, glycerol, thymidine, and tyrosol), and 2 vitamin and other forms (delta-tocopherol and gamma-tocopherol) tended to be reduced in UC patients.

**Figure 3.**
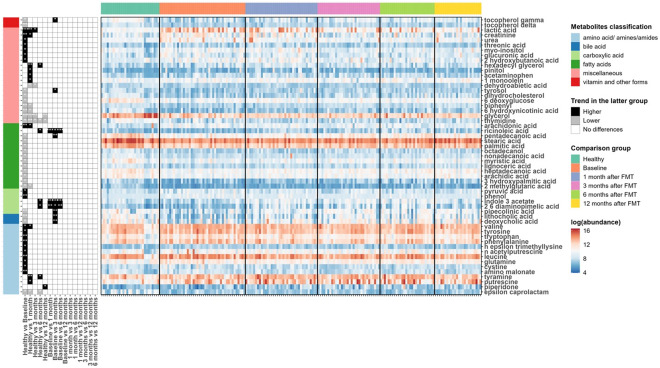
Heat maps showing the log-transformed abundance of differentially abundant metabolites identified by MaAsLin2. Metabolites were grouped according to their classifications (left bar), and the samples were grouped by their phenotypes and time points after FMT for UC patients (top bar). The panel in the left column indicated the coefficients and BH-FDR corrected q values for the coefficients from the linear fixed effects model (Healthy versus UC, with Healthy as the reference group) or linear mixed effects model (UC versus UC at different time points, with UC patients at the previous time point as reference group), with black for higher abundances in the latter group, grey for lower in the latter group, and white for no significant differences between two groups, and * for FDR corrected q value <0.05, ** for FDR-corrected q value <0.01, and *** for FDR-corrected q value <0.001. The panel in the right column showed the log-transformed abundance of differentially abundant metabolites.

Linear mixed-effects models (equation [2]) were fitted to recognize the metabolites with significant changes post-FMT in UC patients. Many metabolites were noted to increase in UC patients after FMT when compared to their baseline metabolomic profiles. Indole-3-acetate, 2,6-diaminopimelic acid, and ricinoleic acid quickly responded to FMT and kept sustained growth for 6 months after one FMT infusion. Deoxycholic acid, lithocholic acid, 3 hydroxyphenyl acetic acid, phenylacetic acid, pipecolinic acid, pentadecanoic acid, 3,4-hydroxyphenyl propionic acid, 3-hydroxybenzoic acid, dihydro-3-coumaric acid, glutaric acid, indole-3-propionic acid, ribose, tyrosol, nicotinic acid, and gamma-tocopherol increased for the first 3 months post one FMT infusion. Phenylacetic acid continued to increase for a total of 6 months after FMT. At 12 months after one FMT infusion, most metabolites in UC patients increased, with the exception of glycerol and thymidine which decreased (Figure 3).

### The effects of FMT were time-limited

PUCAI scores were calculated at baseline and at months 1, 3, 6, and 12 after FMT (Table 2).

**Table 2.** Baseline PUCAI and Endoscopic Mayo scores for 50 paediatric UC patients.

ID	PUCAI	EMS	P1	P3	P6	P12	ID	PUCAI	EMS	P1	P3	P6	P12
1	60	3	50	50	50	50	26	55	2	0	0	0	0
2	60	3	55	50	45	40	27	60	3	25	40	25	25
3	20	1	15	0	0	10	28	55	3	0	0	15	15
4	20	1	15	0	0	10	29	60	3	55	_	_	_
5	30	2	35	30	30	35	30	25	2	10	5	0	5
6	30	2	35	30	30	35	31	40	3	5	5	10	5
7	60	2	0	0	15	0	32	60	3	_	_	_	_
8	50	2	15	10	25	40	33	30	2	50	20	5	5
9	25	1	15	15	0	0	34	50	1	15	5	20	5
10	40	2	30	0	25	5	35	25	2	10	0	15	0
11	60	2	10	10	0	0	36	50	2	0	5	0	5
12	60	2	5	0	0	5	37	60	3	25	5	5	0
13	10	1	0	0	0	10	38	10	1	0	0	0	0
14	35	1	0	0	0	10	39	40	3	15	15	10	25
15	25	3	35	25	35	35	40	30	3	20	20	20	20
16	60	3	40	50	45	40	41	30	3	15	25	30	45
17	30	2	0	10	0	5	42	30	3	20	15	40	10
18	50	3	55	30	30	30	43	40	3	15	25	30	45
19	55	2	20	20	5	0	44	35	3	0	0	0	0
20	20	2	0	15	10	15	45	40	3	0	0	0	0
21	25	3	5	15	5	15	46	20	1	0	0	0	0
22	40	3	45	55	65	0	47	15	1	0	0	0	0
23	40	1	25	10	20	30	48	60	3	0	0	0	0
24	20	2	65	20	20	20	49	30	1	20	10	25	5
25	30	3	5	5	5	5	50	30	1	20	15	40	10

*Note*: PUCAI scores at months 1, 3, 6, and 12 after FMT initiation. EMS, Endoscopic Mayo Score; P1, PUCAI score month 1; P3, PUCAI score month 3; P6, PUCAI score month 6; P12, PUCAI score month 12.

**Figure 4.**
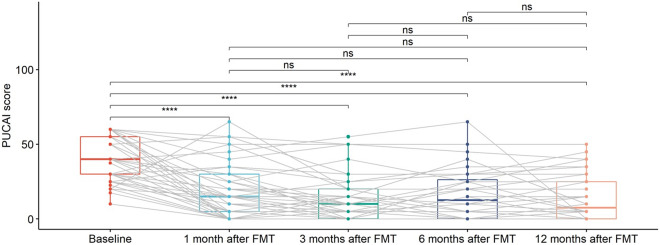
Box plots of PUCAI scores for UC patients at different time points, including baseline, 1 month after FMT, 3 months after FMT, 6 months after FMT, and 12 months after FMT. The grey lines in the figure mark the trajectories of each patient over time. Pair-wise comparisons were performed using paired Wilcoxon rank sum tests, with ns (not significant) for *p* >0.05 and **** for *p* <= 0.0001. Two patients with missing data (UC012 with 3, 6, and 12 months after FMT missing and UC015 with 1, 3, 6, and 12 months after FMT missing) were not included in the analysis.

As shown in Figure 4, there was a statistically significant decreasing trend of PUCAI scores at 1 month after FMT, which continued until 3 months after FMT. PUCAI scores remain flat starting 6 months after FMT. These may imply that the effects of FMT were time limited.

### CDI history, ethnicity, and FMT type influenced the FMT response of paediatric UC patients

**Figure 5.**
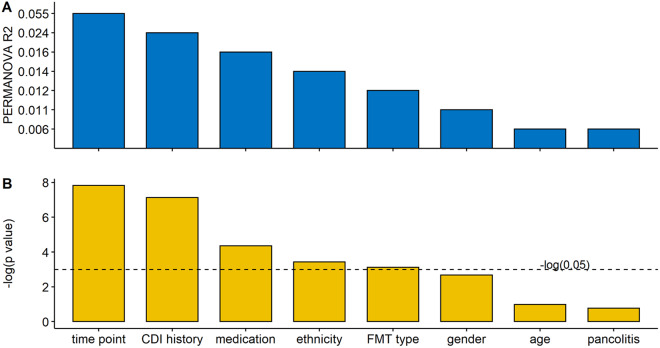
Multivariate analysis showing the amount of inferred variance explained (R2) (A) by each covariate and respective *p*-value (B) determined by PERMANOVA on metabolomics (Bray–Curtis distance on relative abundance). The variance explained by each variable was calculated independently of other variables (the sole variable in the model) to avoid issues related to variable ordering. Time points, CDI history, medication, ethnicity, and FMT type explained a significant but limited fraction of UC patients’ total variation in metabolomics.

Focussing on UC patients, we analyzed the impact of confounding factors such as age and gender. A total of 8 different metadata were collected, including time point, CDI history, medication, ethnicity, FMT type, gender, age, and pancolitis. R2 values and the p-values from PERMANOVA analysis for each variable are shown in Figure 5. Time point had the largest interaction with gut metabolite composition. The other significant confounding factors were CDI history, medication, ethnicity, and FMT type. Medications were not involved in the analysis due to their complicated prescriptions. We fit a linear model (equation [3]) within each time point to determine their influence on the FMT process.

**Figure 6.**
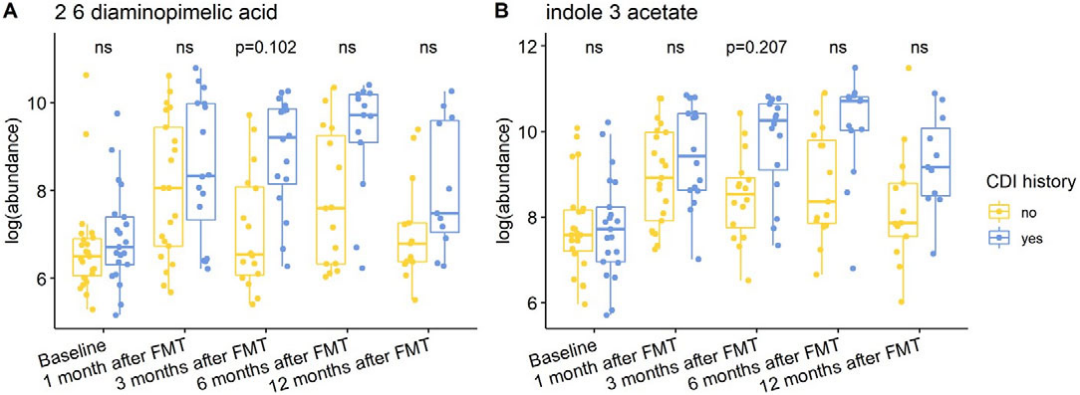
Box plots of the log-transformed abundance of CDI history related metabolites for UC patients at different time points, including baseline, 1 month after FMT, 3 months after FMT, 6 months after FMT, and 12 months after FMT. The differences between patients with and without CDI history were tested using a linear model (equation [3]) within each time point, with ns for not significant, and BH-FDR-corrected q-value annotated.

**Figure 7.**
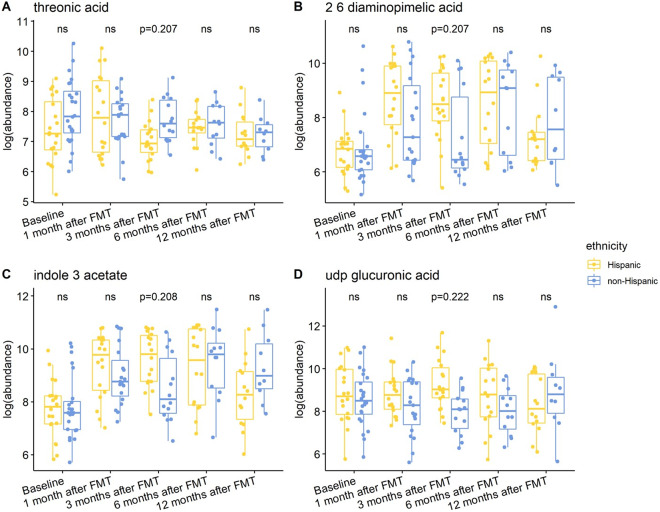
Box plots of the log-transformed abundance of ethnicity related metabolites for UC patients at different time points, including baseline, 1 month after FMT, 3 months after FMT, 6 months after FMT, and 12 months after FMT. The differences between Hispanic and non-Hispanic were tested using a linear model (equation [3]) within each time point, with ns for not significant, and BH-FDR-corrected q-value annotated.

**Figure 8.**
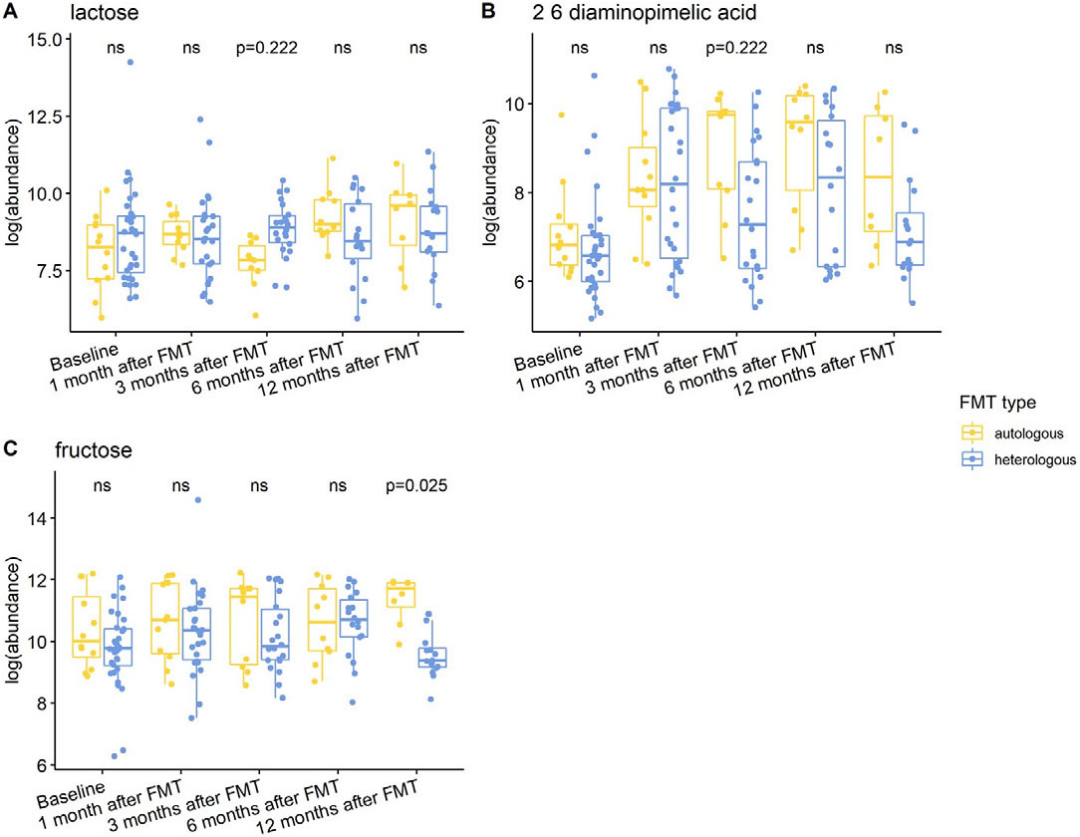
Box plots of the log-transformed abundance of FMT type related metabolites for UC patients at different time points, including baseline, 1 month after FMT, 3 months after FMT, 6 months after FMT, and 12 months after FMT. The differences between patients taking autologous FMT and patients taking heterologous FMT were tested using a linear model (equation [3]) within each time point, with ns for not significant, and BH-FDR-corrected q-value annotated.

Individuals with a history of CDI maintained relatively higher levels of 2,6-diaminopimelic acid and indole-3-acetate post-FMT (Figure 6). The abundance of these two metabolites was also higher in Hispanic patients 3 months post-FMT (Figure 7). Threonic acid was significantly lower in Hispanic patients at 3 months post-FMT. In contrast, UDP-glucuronic acid was significantly higher in Hispanics at 3 months after FMT. Lactose decreased in autologous FMT while 2,6-diaminopimelic and fructose increased in autologous FMT 3 months after FMT (Figure 8).

## Discussion

Gut metabolites are products of microbial metabolism and influence gut health (Sokol, 2020). When comparing microbial profiles of healthy controls to adult IBD patients, studies have found a significant difference and a less diverse gut microbiome (Alam et al., 2020). This decrease in microbiome diversity may result in a shift in the gut metabolomic profile of UC patients compared to healthy patients. Our study found a significant baseline difference in paediatric UC patients’ metabolomic profiles compared to healthy controls. These metabolomic shifts provide insight into the baseline dysbiosis in each UC patient’s gut. Our study also compared the significant difference between 1, 3, 6, and 12 months after FMT and healthy controls. A study done by Moayyedi et al. (2015) was able to show FMT as an effective therapeutic tool in UC patients; however, the data of this study was limited to patients 18 years and older. Our results provide insight into FMT’s potential role in managing UC in paediatric patients.

Most statistically significant metabolites in our study began to trend towards healthy levels within one month post-FMT, and PUCAI scores showed a statistically significant decrease but with a plateau at 6 months. Although there was a continued statistically significant difference at 12 months between the post-FMT and healthy groups, there was a decrease in dissimilarities. This shift towards healthy control levels can indicate a shift in microbiota diversity. This was also illustrated by Moayyedi et al. (2015) within 6 weeks of receiving FMT. If FMT can effectively shift the metabolomic profiles towards healthy donor levels, this could create an environment that allows mucosal healing by reducing inflammation.

### Fatty acids

Previous studies have investigated the role of dietary fatty acids (FAs) in the pathophysiology of IBD (Ananthakrishnan et al., 2014). Our study found eleven FAs that declined in UC patients compared to healthy controls. These FAs included one methyl branched (2-methylglutaric acid), ten saturated long-chain fatty acids (LCFAs) (3-hydroxypalmitic acid, arachidic acid, heptadecanoic acid, lignoceric acid, myristic acid, nonadecanoic acid, octadecanoyl, palmitic acid, pentadecanoic acid, and stearic acid). One polyunsaturated fatty acid (arachidonic acid) was higher in UC patients than healthy controls.

#### Long-chain fatty acids

It has been theorized that the length of FAs plays a crucial role in its effect on gut health, but few have highlighted the role of LCFAs (Ma et al., 2019), defined as FAs with a carbon chain length of 13-21 (Galli, 2009). Our study identified eleven LCFAs of interest in UC patients, including ten saturated and one unsaturated LCFAs. Most statistically significant LCFAs began a trend towards healthy control levels within 1-6 months post-FMT, suggesting that FMT can alter the gut’s LCFA profile.

There has also been debate regarding the impact of saturated versus unsaturated LCFAs in strengthening the intestinal barrier (Benoit et al., 2015). A study done by Benoit et al. (2015) showed that saturated LCFAs, such as palmitic acid and palm oil, enhanced MUC2 synthesis and promoted the differentiation of goblet cells which could be beneficial to intestinal health in IBD. When evaluating the trends of saturated LCFAs, we found that palmitic acid and its derivative 3-hydroxypalmitic acid were declined in UC patients compared to healthy controls.

Arachidonic acid, a polyunsaturated LCFA, was found to be higher in UC patients compared to healthy controls. There have been mixed theories on the role of polyunsaturated FAs in the mucosal health of ulcerative colitis patients. Arachidonic acid has been documented in previous literature to be elevated in ulcerative colitis patients due to the impact of inflammation on the composition of phospholipids in the colonic mucosa (Nishida et al., 1987). Previous literature has also found that monounsaturated fatty acids (MUFA) may play a role in the regulation of gut microbiota and inflammation (Candido et al., 2018). Additional studies have found cis-palmitoleic acid decreases inflammation in the gut of UC patients by increasing the expression of HNF4α and HNF4γ (Bueno-Hernandez et al., 2017).

#### Methyl-branched fatty acid

2-Methylglutaric acid was the only significantly different methyl-branched FA when baseline UC levels were compared to healthy controls. 2-Methylglutaric acid is a metabolite of succinic acid, a citric acid cycle intermediate. This metabolite can be reflective of succinic acid metabolism, which has been hypothesized to play a protective role towards metabolic stress and tissue damage. Excess succinic acid can also play a detrimental role by increasing succinic acid-dependent pathobionts. Overall, succinate is expected to accumulate when the gut is inflamed, so there would be an expected deficiency in 2-methylglutaric acid (Connors and Limbergen, 2019). This theory is supported by our findings of a baseline deficiency with a slow increase post-FMT. This may suggest increased succinic acid metabolism, possibly due to the improved inflammatory state of the gut.

### Amino acids

Amino acids (AAs) have also been theorized to play a role in immunity and the inflammatory state of the gut. Ooi et al. found lower levels of AAs and TCA cycle-related molecules in the colonic tissues of UC patients (Ooi et al., 2011). In our study, ten AAs (aminomalonate, cystine, glutamine, leucine, n-acetylputrescine, n-epsilon trimethyllysine, phenylalanine, tryptophan, tyrosine, and valine) were significantly higher in UC patients than healthy individuals, with only 1 amino acid (epsilon caprolactam) lower in UC patients than in healthy individuals. There has been interest in the role of branched-chain AAs (BCAAs), such as Valine and leucine, which have been linked to modulating the immune response and overall gut health. BCAAs are crucial to produce cells, immunoglobulins, cytokines, and receptors, their elevated levels in UC may impact the inflammatory state of the gut (Nie et al., 2018; Papada et al., 2020).

### Vitamins

Vitamins have been theorized to play a role in intestinal barrier function and modulation of gut microbiota. Tocopherol/vitamin E has previously been linked to protecting intestinal barrier function and modulating the gut microbiota (Liua et al., 2021). Our study found a baseline deficiency in tocopherol with a gradual rise in levels after FMT. These deficiencies may indicate a lack of protective metabolites in the gut of UC patients.

### Secondary bile acids

Secondary bile acids have been theorized to have anti-inflammatory and cytoprotective actions and have been a metabolite of interest as a potential therapeutic option. Our study also found a deficiency in deoxycholic and lithocholic acid at baseline in UC patients, which may suggest a lack of protective effects. Ward et al. investigated the impact of secondary bile acids on cytokine release from colonic epithelial cells. They found that lithocholic acid potently inhibited epithelial cytokine release and protected against mucosal inflammation (Ward et al., 2017).

### Nucleosides and nucleobases

DNA nucleosides, specifically thymidine, and their impaired incorporation into DNA have previously been linked to colitis regardless of disease severity (Alpers et al., 1980). However, our study found that at baseline UC patients were deficient in thymidine.

## Conclusion

This study showed that baseline metabolite profiles in UC paediatric patients are different from healthy subjects. Additionally, we demonstrated that FMT alters metabolite profiles. Our data suggested a time-dependent trend from UC type to healthy profiles after FMT. FMT may be effective in altering the inflammatory state of the gut by increasing the abundance of anti-inflammatory metabolites and decreasing pro-inflammatory metabolites. Future studies should investigate the timing and need for repeat FMT and follow their trends. Overall, our study was notable for a significant baseline difference in the metabolomic profiles and a significant difference at follow-up months 1, 3, 6, and 12 when comparing UC to healthy controls. These differences were primarily seen with FAs, AAs, nucleosides/nucleobases, vitamins, and bile acids. Further studies with larger sample populations are needed to identify significant differences in specific metabolites and their trends post-FMT.

## Limitations

Limitations of the study include the inability to control for patient’s environment and diet at home, loss of data points due to loss of follow-up, and insufficient data on very early-onset ulcerative colitis.

## Data Availability

Details regarding access to data, materials, protocols, and software will be made available upon request.
